# A mixed methods study of self-directed learning in clinical practice using a mobile skills training system

**DOI:** 10.1186/s12909-025-08127-1

**Published:** 2025-10-29

**Authors:** Marie-Louise Södersved Källestedt, Patrik Hidefjäll

**Affiliations:** 1 Centre for Clinical Research, Uppsala University, Mälardalen University, Västerås, Region Västmanland SE-72189 Sweden; 2 Affiliated to School of Health, Care and Social Welfare, Mälardalen University, Box 883, Västerås, 72123 Sweden; 3 Unit for Bioentrepreneurship, Karolinska Institutet, Stockholm, SE-17177 Sweden

**Keywords:** Self-directed learning, Continuing professional development, Healthcare professionals, Skills training

## Abstract

**Background:**

The challenge of increasing demands on healthcare and a less available workforce requires new approaches to retain and develop healthcare professionals. One way of addressing this challenge is by instituting competency-based continuing professional development based on self-directed learning (SDL) principles. We investigated the feasibility of a mobile training system in a clinical and peer-to-peer, SDL setting.

**Methods:**

The study used mixed methods with surveys, observations, and interviews to gain comprehensive insights into how healthcare professionals (nurse, assistant nurse) and managers experienced the investigated educational tool.

**Results:**

Healthcare professionals’ experiences of SDL were illustrated in four main themes: (1) leadership required for learning, (2) conditions for learning, (3) effects of continuing professional development, and (4) suggestions for improving training methods. In surveys, healthcare professionals self-assessed their confidence in executing care tasks before and after undergoing SDL for those tasks. The results indicated a significant increase in self-efficacy post-SDL training. Engaging in care tasks through SDL, which includes checklists aligned with evidence-based practices, enhances healthcare professionals’ awareness of their own competence. Additionally, it provides managers with the means to ensure that employees meet core competence requirements. The role of a clinical skills centre to support managers and staff in their learning efforts was seen as important for sustained learning.

**Conclusion:**

A mobile training system, implemented within a clinical and peer-to-peer SDL framework, facilitates and complements competency-based continuing professional development and reduces the perceived length of time healthcare professionals spend away from direct patient care. Access to clinical educators for guidance during implementation, along with support from the clinical skills centre during the planning and follow-up stages, proved essential for facilitating accessible learning. Managers play a crucial role in prioritizing continuous learning for their employees and establishing a culture of ongoing education among managers and healthcare professionals.

**Supplementary Information:**

The online version contains supplementary material available at 10.1186/s12909-025-08127-1.

## Background

The challenge of increasing demands on healthcare and less available workforce requires new approaches to develop and retain critical professional groups in healthcare such as nurses. One way of addressing this twofold challenge is by instituting a competency-based continuing professional development (CPD) program that supports self-directed lifelong learning in a clinical practice setting. A competency-based CPD views competence as a dynamic process in the context of the changing demands of a practice, shifting the focus from attending standardized courses to demonstrating practical application of skills in real-world clinical scenarios. It emphasizes self-directed learning (SDL) processes and promotes the role of assessment as a professional expectation and obligation [1]. SDL is defined as “a process in which individuals take the initiative, with or without the help from others, in diagnosing their learning needs, formulating goals, identifying human and material resources, choosing and implementing appropriate learning strategies, and evaluating learning outcomes.” [2, p. 18]. SDL principles address that adult learners need self-direction based on experiences, current needs and interests [2, 3].

The early initiatives in continuing professional education were grounded in the belief that SDL was the dominant and most effective mode of learning for healthcare professionals. It was also assumed that SDL depends on peer-resource groups, guidance, and other resources to be successful [3]. Since then, many studies of how a CPD program is implemented in various healthcare systems have pointed to several problematic aspects such as lacking organisational commitment, financial limitations, administrative barriers, limited time for healthcare professionals to participate and remoteness to actual clinical practice and patients that make CPD less accessible and effective [4]– [5]. A more recent review of CPD programs in healthcare highlighted that most CPD activities lack strategies to support the implementation of learning into practice [6]. Furthermore, the needs for CPD also change over a nursing career [7].

Although there are different challenges and barriers to CPD, there is also a set of common factors that enhance its impact. A key success factor tends to be how well educational activities adapt to the current workload; crucially, educational efforts in clinical practice should support, rather than interfere with, patient care [4]. Upholding a sufficient nurse staffing level has been shown to be critical for patient outcomes and safety [8, 9]; therefore, educational activities cannot be allowed to interfere with the core task of patient care. Strong support from both educators and management is essential for embedding CPD into the workplace and making learning an integral part of the workplace culture. This support helps ensure that learning activities are relevant to clinical practice and, in turn, improves nurses´ self-motivation to engage in CPD [10].

Self-efficacy in clinical performance plays an important role in the application of competence, much like the supportive environment described above fosters engagement. Previous studies have shown that education is crucial for home care staff’s self-efficacy in contributing to self-care in heart failure [11]. Similarly, a study emphasizes the importance of nursing students being aware of their personal factors and self-efficacy, which in turn can influence their performance in clinical settings [12].The need to adapt CPD to clinical practice conditions obviously creates limitations on how to design educational activities that more flexible, mobile e-learning, peer-to-peer learning, and simulation methods may be able to address [13–15]. A mobile e-learning platform that combines simulators (mobile skills trainer), evaluation apps on mobile phones, and an administrative backbone to manage training activities that take place in a distributed way at the workplaces of healthcare professionals, may be a feasible way to meet those constraints [16].

The purpose of the present study is to investigate the feasibility of such a mobile training system in a clinical and peer-to-peer SDL setting. This way of learning may provide opportunities for collaboration, reflection, and sharing of experience among practitioners in their workplace. We, therefore, address the following questions:

1. How do healthcare professionals experience learning with a mobile skills training system, when used in a clinical and peer-to-peer SDL setting?
2. What experiences do managers have of healthcare professionals learning in their daily work and of possible effects of learning on the employee, the workplace, and/or the patient?

## Method

This study evaluates a new mobile tool to support self-directed peer-to-peer learning in a hospital care setting. The study included healthcare professionals working at a mid-sized Swedish regional hospital serving both the immediate urban and rural areas.

### Study design

The study used mixed methods with surveys, observations, and interviews in order to gain comprehensive insights into how healthcare professionals and managers experienced the investigated educational tool https://laerdal.com/information/simcapture-for-skills/.

This mixed-methods study employed an exploratory/explanatory sequential intervention design, in which participants completed a survey before and after using the mobile skills training system (Supplemental material 1). The results of participants solving critical clinical tasks were observed using checklist-based questions in the mobile skills training system. The quantitative results could then be further contextualized and embedded by follow-up interviews.

### Context

The following care tasks were available for training: (1) cardiopulmonary resuscitation (CPR), (2) tracheostomy care, and (3) urinary catheterization. Most of the healthcare professionals practiced CPR (62.8%), followed by tracheostomy care (20.9%), and urinary catheterization (16.3%). Skills training with CPR contained eight parts plus a part about applying a pharyngeal tube correctly; the urinary catheterization contained 14 parts and tracheostomy eight parts.

Educational coordinators, employed at the clinical skills centre of the hospital, are appointed to facilitate CPD and learning of healthcare professionals in their clinical practice, thus functioning as a resource in the SDL [2, 3]. Participants conducted observations in pairs using checklists integrated into the system, with one peer documenting their colleague’s performance. The authors then statistically analyzed the quantitative results from these observations.

Educational coordinators created checklists following national and/or clinical guidelines in the SDL program (Supplemental material 2). The SDL program used was SimCapture for skills from Laerdal (Laerdal Medical AS, Stavanger, NO), named mobile skills training system in our study. Clinical educators borrowed training materials from the Clinical Skills Centre to their own department. Typically, the department had access to the training materials 24 h a day for a duration of two weeks. Educational coordinators provided support to the clinical educators. During the training sessions, healthcare professionals practiced in pairs, engaging in peer-to-peer learning. They alternated roles, taking turns as performers of care tasks and facilitators using the system checklist. The completed checklist provides information about how the participating healthcare professional performed the care task as observed by a peer.

### Data collection

Data were collected from surveys taken before and after the skills training. Our sampling strategy combined cluster sampling, selecting key departments within the hospital, with convenience sampling. Within each selected department, participants who volunteered were included in the study. Seventy-eight nurses or assistant nurses voluntarily responded to an invitation to participate in the surveys (only healthcare professionals participating in the survey both before and after SDL activity were included, ending up with response rate 77%). The survey covered the following areas: (1) experience of self-efficacy, (2) SDL as a part of professional practice, and (3) experiences of the educational environment and the learning method. The healthcare professionals self-assessed their ability in the survey.

Observations of SDL were made during the period from January 2022 to January 2023 by 78 nurses and assistant nurses. One physiotherapist showed up and wanted to participate, this person was allowed to try SDL but was excluded from the study. Observations of the SDL occurred where two healthcare professionals took turns to perform the care tasks and be the facilitator/observer (peer-to-peer learning). What the facilitator observed was recorded according to checklist questions in the mobile skills training system. The first author participated in about 30 of the events where a care task was performed, the second author observed one subsequent training event, and all the observations were stored in the mobile skills training system.

The qualitative data were collected from interviews with the target groups: assistant nurses including clinical educators (n 6); nurses and assistant nurses including clinical educators (n 8); and educational coordinators (n 2); and their managers (n 5) (See Table 1), with previous experience of working as registered nurses. All participants in the interviews were female except for one male. To enhance transferability, participants were recruited from diverse wards with different specialisations such as medicine, orthopaedics, surgical, intensive care, and anaesthesia involving adult patients.

**Table 1:** Characteristics of the included healthcare professionals

Characteristics		Interviews (N=21)	Observation & survey (N=78)
Gender	Female	20	64
	Male	1	14
Age	19–30 years	7	13
	31–40 years	3	21
	41–50 years	9	26
	51–60 years	2	20
Profession	Nurse^a^/Clinical	7	48
	educator^b^		
	Assistant nurse^c^/Clinical educator	9	38
	Manager^d^	5	
Number of years worked in healthcare	1–5	4	18
	6–10	8	12
	11–15	2	15
	16–20	2	15
	>20	5	26
Interview	Face-to-face^e^	10	
	Distance	10	
	Interview minutes	27 (9–44)	
Observation/care task	Performed >once^f^		23
	CPR		53
	Trak		14
	U-kat		18

^a^One nurse participated in the interviews and had trained through SDL

^b^Ten internal educators including two educational coordinators participated in the interviews. They had tested training through SDL and are trained to help healthcare professionals if technical problems should occur. Six of the internal educators are registered nurses

^c^Four assistant nurses participated in the interviews and had trained through SDL. Four of the internal educators are assistant nurses

^d^All managers are registered nurses and have previously worked as nurses

^e^Interview place chosen by the informant, face-to-face or distance meeting by telephone/digital video conference

^f^Number of healthcare professionals performing SDL more than one time

Interviews were conducted according to a predefined interview guide. Interviews were performed from September 2022 to January 2023, by the first author. An interview guide was pilot tested and thereafter used in all interviews to enhance reliability. No change to the draft interview guide was needed. The opening question was: “*I would like you to start by telling me about your experiences of learning in everyday work*.” Subsequent questions addressed the following themes: (1) Learning in everyday work; (2) What experiences do you have of learning with skills training through SDL; (3) As a manager, healthcare professional or clinical educator, how do you experience the user functions and the learning method and system, please describe; and (4) What opportunities does learning provide for healthcare professionals immediately before seeing a patient?

Face-to-face or digital remote interviews (average duration of 27 min, minimum nine minutes to maximum 44 min) were performed, and audio recorded. In some of the departments, leadership was shared between two managers, meaning that both were jointly responsible for the department and its staff. As a result, some interviews included both managers participating together.

### Data analysis

Only participants who completed both surveys and participated in the observation were included to ensure integration of results by a mixed methods embedding approach [17].

#### Survey

Descriptive statistics were used to summarize data collected from the surveys. The surveys revealed a statistically significant shift in responses along the scale from ‘not true’ to ‘true’, as determined by the Wilcoxon test and assessed using the chi-square test, with a significance level of *P* < 0.05. This analysis enabled the identification of meaningful changes in healthcare professionals’ skill levels during the learning process.

#### Observation

Healthcare professionals learning a specific care task through SDL were grouped into three categories: those who fully performed/approved, partially performed, and those who failed to perform it correctly. Descriptive statistics were applied to the observational data. The Wilcoxon test, assessed using the chi-square test, *P* < 0.05), was used to identify statistically significant changes in observed performance. This provided a complementary perspective to the survey findings, offering insights into actual behavioural outcomes during the skills training.

#### Interviews

The interviews were transcribed and managed using Nvivo (version 1.4) through thematic analysis, using a data-driven inductive approach [18, 19]. Nvivo was not used as an analytical tool in the thematic analysis, but rather as a means to organize and manage the data material. Transcriptions were thoroughly read for understanding, and important sections were identified, coded, and grouped into themes based on similar meanings. Potential themes and subthemes were pinpointed and refined to create clear definitions and labels. To ensure analytical credibility, the first and last author reviewed and discussed the content multiple times. The results were translated into English, reviewed by a language professional, and compared with the Swedish version. Any discrepancies were addressed through back-and-forth reviews, confirming the results.

### Ethical approval

The Swedish ethical review authority in Uppsala (Dnr 2020–05591) approved the study and the principles of the Declaration of Helsinki [20] (General Assembly of the World Medical Association, 2014) were followed. Participants gave written informed consent to participate in the study.

## Results

The experiences of healthcare professionals’ SDL were summarized into four themes, based on a thematic analysis of the interviews: (1) Leadership required for learning, (2) Conditions for learning, (3) Effects of CPD, and (4) Suggestions for improving training methods. Each theme, its subthemes, and codes are illustrated in Supplemental Material 3 and 4.

The theme “Effects of CPD” (3) was investigated using a mixed-methods approach that combined interviews, survey and observation.

The healthcare professionals that participated in the survey and observational part of the study were nurses (55%) and assistant nurses (44%), Table 1.

### Leadership required for learning

This theme describes healthcare professionals’ experiences of leadership needed to foster learning and the importance of blending online education with SDL skill development. Interviews clearly showed that managerial expertise and commitment are crucial forestablishing a well-functioning and supportive learning structure. This includes the strategic appointment of clinical educators to lead and organize workplace learning.

#### Responsibility for creating a learning culture

Perceiving learning as an integral part of care has been a dominant theme in the interviews of managers, though with challenges in facilitating the learning process. A related issue was who can make informed decisions regarding the content of the learning initiatives and when to implement them. The managerial perspective emphasized the consistent prioritization of patient care above all else. “*The important thing is to take care of the patients*,* but actually*,* without education and learning we would not have been able to do that in a good way*.” (Interview 20, manager).

It became evident that managers who prioritize learning as an integral part of the daily work create a culture of learning. *‘If I put it (learning) in focus and say*,* ‘This is something we must do’… It becomes such a culture in a workplace if I also prioritize it (learning)*.‘” (Interview 17, manager).

Managers understood they have the ultimate responsibility for ensuring that their employees are prepared by having the required competence to carry out the work. This responsibility to ensure that employees are up to date regarding their skills was expressed as sometimes burdensome. On the other hand, most employees were aware of their own responsibility to develop and maintain their competence to do a good job. “*I think a lot also lies in personal responsibility. Everyone has a personal responsibility to improve and keep up with developments in care practice*,* and then*,* of course*,* the Region* (the responsible authority and owner of the hospital) *also provides valuable support. One needs to develop one’s competence*,* learn mor*e *and be open to it.”* (Interview 6, participant).

The inclination towards CPD became evident when managers delegated the responsibility to employees to actively contribute to their colleagues’ learning. This was facilitated by appointing healthcare professionals specifically for this role. *“Managing all training alone becomes too challenging for a manager. It is therefore crucial to have healthcare professionals who take initiatives and genuinely believe in the value of this endeavour. This ensures that it doesn’t become overwhelming for everyone*,* including ourselves*,* and that we don’t lose momentum.”* (Interview 3, manager).

The clinical educators were aware of the essential procedures that needed to be practiced and rehearsed. Those clinical educators who were assigned specific areas of responsibility took pride in their mission. *“Yes*,* the result of having contributed to organizing learning makes me proud of myself (laughter].”* (Interview 2, clinical educator).

The observed outcome indicated that fostering a culture of learning was achievable when there was an expectation for healthcare professionals to attend seminars, obtain certificates, engage in daily reflection sessions, and undertake responsibilities such as supervising students and colleagues. Further, the observed outcome was that when education was deliberately integrated into the schedule, a cascade effect ensued, prompting active participation and engagement from all healthcare professionals and colleagues.

Managers and clinical educators expressed their need for assistance in both planning and follow-up of learning exercises and the crucial role of the clinical training centre in providing this support.

#### Integrating learning into clinical practice

There were a set of challenges to establish a good learning environment and routines that harmonize with clinical practice. Several learning methods complement each other, and it is described as challenging to facilitate their use. Online learning with certificates seemed effective for healthcare professionals to start with, allowing them to assess theoretical knowledge before engaging in practical skills training through SDL. However, a challenge associated with online learning was the temptation to merely click through answers without genuinely absorbing the content. *“My experience with competency cards (online learning) is that they are very good. But it’s also up to oneself because it’s possible to cheat through them*,* unfortunately.”* (Interview 13, clinical educator).

The observed scenario revealed challenges in organizing practical skills training prior to the introduction of SDL. While online learning was beneficial, healthcare professionals emphasized the importance of supplementing it with hands-on skills training, stating: *“It still needs to be taken into practice. Yes*,* of course*,* digital training is good*,* but I think it’s just ticking off the boxes*,* not learning much from it. So*,* it really needs to be taken into practice.”* (Interview 6, participant).

It became apparent that online learning with certificates and SDL mutually reinforce each other. Certain caregiving tasks required training to ensure they could be executed proficiently in any situation, irrespective of circumstances, *“even blindfolded if needed”*. (Interview 10, manager)

However, for this mutual reinforcement and successful integration to occur, it was necessary for someone to take on the responsibility of incorporating SDL into daily work routines, as a complement to other learning methods, such as support from a clinical educator, as emphasized in the following quote: *“It really requires someone who is like*,* ‘now let’s take hold of it and do it.‘”* (Interview 13, clinical educator) It was expressed that the manager holds the overall responsibility for integrating learning into everyday work.

Concerning SDL in the field of cardiopulmonary resuscitation (CPR), clinical educators observed that the presence of an instructor was necessary for quality assurance during CPR procedures. *“The quality is very difficult to ensure. They don’t assess each other maybe in the way the instructor assesses. You can see that ‘you’re not releasing properly’ or ‘you’re not doing the compressions properly*,*’ ‘the breaths are not as they should be*,*’ even though peers might not say that to each other.”* (Interview 8, clinical educator).

There were concerns about the facilitator in SDL marking a step as completed when in reality it was not, often due to colleagues hesitating to speak up. Certain care tasks showed more success in learning through self-directed methods using this specific program than CPR.

In summary, healthcare professionals viewed SDL as a valuable complement to online learning. Designated clinical educators played a crucial role in ensuring high-quality learning experiences. According to managers, having senior nurses on the ward to support their colleagues would be ideal. SDL facilitated the assurance of a baseline common competence level.

### Conditions for learning

This theme describes the experiences of healthcare professionals regarding conditions necessary for meaningful learning. Interviews and observations emphasized that having dedicated time and space for learning were essential for creating optimal healthcare environments that support learning. Clinical educators were instrumental in creating structure and making learning accessible at all hours of the day.

#### Prioritize learning/training

Managers emphasized the need to prioritize learning, as it contributed to the attractiveness, motivation, joy, and pride of healthcare professionals. *“There’s hardly ever enough time for everything*,* but it’s essential to make time. Given my prioritization of education and developmental matters*,* it’s evident that education must take precedence. It not only nourishes us but also fosters increased motivation*,* attraction*,* curiosity*,* joy*,* and pride.”* (Interview 10, manager).

Experiences varied concerning how time for learning was utilized and prioritized. While everyone recognized the importance and necessity of training, various challenges could hinder its timely implementation. The consensus was that if training was integrated into the schedule, learning became achievable; otherwise, it tended to be overlooked. SDL was perceived as a valuable method, facilitating learning at any time of the day. This was particularly helpful since there are no specific natural times in the day when training can be easily scheduled.

Not only time, but also aspects related to accommodation were considered as important prerequisites for meaningful learning to occur. It was noted that it was crucial to arrange separate training sessions in a secure and secluded environment to mitigate potential anxiety. *“We stood in the wrong place. We stood in a very open area*,* which I also think people found a bit challenging. Maybe it didn’t turn out so well.”* (Interview 8, clinical educator).

#### Organize training

Clinical educators found that making learning accessible involved allowing healthcare professionals to influence the pace and quantity of learning they required.

*“Yes*,* but everyone is unique*,* some may need to practice ten times*,* while others may only need to do it twice. It’s crucial to identify the factors hindering learning. Why haven’t they grasped this? Are there personal barriers*,* nervousness*,* or fear? Understanding these aspects is essential for individual customization*,* and it holds significance.”* (Interview 18, clinical educator).

Clinical educators played a key role in providing support and safety during skills training for healthcare professionals. *“Yes*,* she was only there to provide support. But she wasn’t involved in assessing how we carried it out.”* (Interview 16, participant).

Clinical educators who rotated between various workplaces and worked flexible hours throughout the day contributed especially to the learning process. *“Then there was a guy who had done self-directed learning during the day*,* and the following week*,* he was going to work the night shift. So*,* he helped them get started and was available for questions*,* allowing the night staff to practice as well.”* (Interview 11, clinical educator) Participants perceptions were that healthcare professionals working during the night often went unnoticed or were easily overlooked.

Clinical educators expressed apprehensions that the introduction of SDL may lead managers to deprioritize the allocation of time for organizing and conducting practical training sessions. *“I think the purpose of self-directed learning from the clinical management has been to reduce the time that we instructors spend on our training. So*,* if some group of colleagues would need an instructor*,* it’s not certain that we’re allowed to leave (to organize for them).”* (Interview 9, clinical educator).

The educational environment and learning method indicated a positive outlook. The information and support function from the clinical skills centre in organizing training material was seen as helpful.

### Effects of continuing professional development

This theme describes the learning effects of Continuing Professional Development (CPD), as identified through interviews, observations and surveys. It became evident that engagement in SDL activities led to healthcare professionals developing a heightened awareness of their own competence.

#### Relevance for the individual

Healthcare professionals had a positive experience with how they received feedback on their learning from the system and engaged in collaborative peer feedbackThis process boosted their awareness of their own competence through SDL training and perceived self-efficacy. The peer-to-peer nature of this learning method fostered mutual support, and the program’s use of numerical data ensured feedback was not seen as personal criticism. As one clinical educator shared, *“I think it feels very appropriate to be in pairs. You can get good help from each other. It leads to good discussions… or well*,* reflections and feedback. Now you have something to lean on a bit more*,* so concretely.”* (Interview 11, clinical educator).

Engaging in pair learning through peer-to-peer interactions prompted healthcare professionals to reflect on their own task execution in relation to their colleagues during care situations. This collaborative reflection process contributed significantly to the overall learning experience. “*So*,* the other one (my colleague) got to go first. And it was also interesting to see how she did it. And then you immediately felt*,* ‘yes*,* but no*,* that’s not how I do it*,* or… And if that was correct*,* then I must be doing it wrong.‘”* (Interview 4, participant).

Practicing SDL in pairs created a relaxed atmosphere. *“Being with a colleague… made you feel a bit safer too. And that you… that you were just sort of in pairs.”* (Interview 15, participant).

#### Results from survey

In the survey, healthcare professionals responded to questions regarding their perceptions of self-efficacy, learning, and leadership in care situations both before and after undergoing skill training in SDL. Following their skill-trained care situations with SDL, the healthcare professionals self-assessed their self-efficacy as higher than before using this learning method (see Table 2), with more “true” answers after training. The Wilcoxon test yielded *p* < 0.017. It also emerged during the analysis that more healthcare professionals fully or partially agreed that they have the ability to lead the work close to the patients, and further involve the patients in the care tasks (see Supplemental material 1). When comparing self-efficacy outcomes of completed care tasks, no statistically significant difference was identified in the perceived self-efficacy between healthcare professionals who were deemed competent – i.e., those who successfully performed the care task in accordance with the evidence based checklist – and those who were not, referring to healthcare professionals who failed to meet the checklist criteria.

**Table 2:** Perceived self-efficacy before and after self-directed learning

	^Before^	^After^
Not true	^5 (6.4)^	^3 (3.8)^
Partly true	^25 (32.1)^	^13 (16.7)^
True	^48 (61.5)^	^62 (79.5)^
Total N	^78 (100)^	^78 (100)^

Cross tables of self-assessments displaying changes before and after self-directed learning. Changes are read row-wise left to right, e.g. participant reporting “not true” before practicing and how they report after practice

Professionals found training with a peer beneficial and perceived the training material and time as well-suited for self-directed skills training. They found the information provided before training to be sufficient, and healthcare professionals self-assessed their capability to apply acquired knowledge when interacting with real patients as high. Furthermore, a greater number of healthcare professionals considered learning to be an integral part of their professional practice after training with SDL. (see Additional file 1)

#### Results from observations

Results from the mobile skills training system revealed no distinct correlation between the duration of healthcare professionals’ experience in the field and their performance in skills training. For those who chose to practice the same care task twice, it appeared that they increased their ability to perform the task (see Table 3).

**Table 3:** Approved care tasks (%) from first to last skills training with self-directed learning

	Results from first skills training			Results from the last skills training		
Category	Frequency (n)	Percent %	*p*^b^	Frequency (n)	Percent %	*p*^b^
*Failed*	19	24.3		3	13.0	
*Partly performed*	13	16.7		4	17.4	
*Approved*	46	59		16	69.6	
Total	78	90.7	< 0.001	23	100	<.01^c^
Missing^a^	8	9.3		0	0	
Total	86	100.0		23	100.0	

^a^Missing due to incomplete self-assessment

^b^*p*-value from Chi-square test

^c^More healthcare professionals performed the care task correctly after training more than once

For those practicing CPR, correct execution ranged from 56% to 100%, with the assessment of vital signs being the least accurately performed.

In SDL using the tracheostomy care skills module, healthcare professionals performed tasks accurately within the range of 67%–100%. Drawing air out of the cuff with an empty syringe was the least accurately performed task. In current urinary catheterization, professionals executed tasks correctly at rates ranging from 87 − 100%, with the identity check of the patient and thorough hand washing and disinfection being the least accurately performed.

#### Relevance for the patient and the workplace

Clinical educators placed high demands on themselves to execute care tasks in strict adherence to guidelines and evidence-based practices, ultimately benefiting patients. Those who engaged in facilitating learning displayed a profound passion for their own educational journey and expressed a genuine desire to contribute to their colleagues’ ability to provide safe and proficient care.

Healthcare professionals affirmed that their colleagues felt more confident when practicing on training models. As one educator highlighted in an interview, *“People feel more secure practicing on a mannequin first*,* completing all the steps before transitioning to human patients.”* (Interview 13, clinical educator).

The prevailing experience suggested that patients required healthcare professionals who exude confidence in meeting and caring for their healthcare needs. *“So*,* this (self-directed learning) makes us safer and more secure. Yes*,* and what does a patient need*,* a sick person needs… nothing but a safe and warm and knowledgeable staff around him. And I think these simplistic learning methods are helping us*,* so I´m really happy”.* (Interview 10, manager) It was also noted that learning within one’s own workplace contributed to a sense of safety. This safety, in turn, established the necessary conditions for a safe interaction with the patient. *“So*,* I think that the biggest benefit for the patient is that you are confident and secure. And you know that if you get a question… You should be able to answer questions if the patient asks you*,* ‘why are you doing it this way?’ It has to be professional; you know. So*,* there are only advantages to it (self-directed learning).”* (Interview 17, manager).

The qualitative interview findings aligned with the survey results, indicating consistency in healthcare professionals self-assessed self-efficacy (see Results from Survey). Moreover, the interviews revealed that the reflective process with peer-to-peer learning in the SDL was perceived as less intimidating, which had positive.

implications for workplace learning and professional development. *“You stand and reflect at the same time*,* two colleagues who are learning. It’s also neat and holds great significance for learning. And it helps to de-dramatize.”* (Interview 14, clinical educator) Furthermore, managers expressed the belief that the SDL method could streamline the introduction process, reduce time away from patient care, enhance quality assurance, facilitate competence transfer, and support delegation. *“So*,* also getting help with delegations. Being able to do this kind of self-directed learning adds a bit more certainty*,* at least.”* (Interview 3, manager). The chosen learning method also played a contributing role; *" To ensuring that everyone performs a task in the same way*,* that it is evidence-based*,* and that it is based on a platform where the information is derived from evidence-based knowledge.”* (Interview 11, clinical educator).

The interviews revealed experiences of prioritizing peer-to-peer learning and the exchange of experiences between new graduates and senior professionals, enhanced daily learning. This form of learning was noted to instil a sense of capability in patient care situations. *“We never know what we have to take care of*,* but we have each other*,* and yes*,* that is a comfort.”* (Interview 10, manager).

### Suggestions for improving SDL

This theme describes the improvements that emerged through interviews as desirable and also highlights the need for clinical educators to be given the opportunity to develop.

#### Technical and communication development

Concerns had been raised regarding the use of the mobile skills training system, with participants expressing that it would be more convenient if all text were in Swedish, rather than a mix of Swedish and English. A suggested improvement was to include an option for text-to-speech. As one participant noted, *“I learn better*,* for example*,* if… when I’m on the internet reading*,* I highlight everything*,* and then I have it read aloud. I think*,* since there are many with dyslexia. Am I going to read all of this?”* (Interview 7, participant) The suggestion was to provide the option of having the content read aloud for individual users.

Managers also wanted the ability to view their employees’ results in the system at a group level.

Furthermore, a valuable enhancement for system administrators would be Another valuable enhancement for system administrators was to simplify the process of implementing new learners’ names. The current method, which involves using of an Excel file for import, was not considered user-friendly for healthcare professionals, the primary target group.

#### Empowering clinical educators to enhance learning

In the analysis of the interviews, the clinical educators were central in making the learning method meaningful and motivating for other healthcare professionals. By focusing on the development of clinical educators — providing them training within the system, granting access to educational materials, and offering support from managers and system super users to facilitate and organize learning sessions — both they and other healthcare professionals can experience even greater improvement in their learning processes.

## Discussion

The purpose of the present study was to investigate the feasibility of a mobile skills training system and to explore how healthcare professionals’ experienced learning through its use based on principles of SDL. In addition, the study examined the experience of managers and aimed to identify potential effects on the individual, the workplace, and/or the patient.

Our study highlighted the flexibility of SDL for healthcare professionals. They could tailor their training to their specific needs and choose the most convenient time and space for them in combination with other learning methods. To create comprehensive CPD, it is essential to get learning as a part of the daily work, as suggested by Koskimäki and colleagues [21]. What was unique about our study were the different perspectives of healthcare professionals, clinical educators, and mangers that all suggested that online learning, coupled with a certification, was beneficial for reviewing theoretical knowledge before engaging in hands-on skills training through SDL. Other references also support the idea that online education serves as a good preparation method [22, 23] and that mixing methods for learning is beneficial for the learners [24].

The combination of interviews and observational data provided a comprehensive understanding of how different learning methods support skill training. Interviews revealed that healthcare professionals appreciated the immediate feedback provided by the mobile skills training system, as well as the collaborative peer feedback. The peer-to-peer nature of this learning environment fostered mutual support, and the use of numerical performance data helped ensure that feedback was perceived as constructive rather than personal criticism. Peer-to-peer learning encouraged reflection on individual task execution in relation to colleagues, enhancing the overall learning experience.

However, when it came to CPR training, findings from both interviews and observations indicated that self-directed learning (SDL) alone was insufficient. Clinical educators emphasized the necessity of instructor presence to ensure quality assurance during CPR procedures. Observational data from the mobile training system further supported this, showing that CPR had the lowest percentage of correct performance among all practiced clinical skills. This suggests that while SDL and peer learning are effective for many skills, CPR training requires structured guidance and real-time supervision to maintain patient safety and procedural accuracy. The peer-to-peer learning was also supported in other studies [25] that also highlighted that expert led training is important.

Our study highlighted that the way learning was organized forms the foundation for healthcare professionals to develop their skills. Clinical educators played a crucial role, proven to be a learning resource that contributed to a culture where healthcare professionals voluntarily felt a need and desire for CPD. Managers, in turn, established the groundwork for a learning culture in the workplace. Continuous support from educational coordinators at the clinical skills centre, coupled with the integration of real clinical practice made learning easily accessible for healthcare professionals. Other studies have also pointed out the importance of guidance and other resources [3], where the important “other resources” in our study proved to be clinical educators. These resources in combination with committed managers can solve what others [4–6] have seen as problematic aspects such as a lack of organizational commitment and remoteness to actual clinical practice and patients, thereby enhancing CPD among healthcare professionals. Mlambo et al. (2023) calls for organizational strategies to facilitate CPD [5]; we suggest that clinical educators play a crucial role in this process. Clinical educators organize, follow up, and provide opportunities for reflection and feedback, allowing colleagues to demonstrate their competence based on evidence. Peer-to-peer learning with colleagues initiates reflection and feedback, further supported by clinical educators. They contribute to maintaining and fostering a social learning culture by addressing challenges in everyday work and learning to support colleagues’ CPD. Justus et al. (2016) provided evidence that clinical educators working in healthcare and being engaged in learning are associated with fewer urinary catheter infections [26]. The mobile skills training system used in our study allows identification of areas in care tasks where healthcare professionals may deviate from guidelines.

Peer observations complemented the interviews, revealing that multiple repetitions of skills training led to an increased approval rate of care tasks—an outcome that, while seemingly intuitive, has now been empirically demonstrated by this study. The authors’ observations provided crucial insights for interpreting the interview and observational data.

At group level, a low percentage of approved care task when performing skills training, could indicate a need for additional learning methods. In line with Farnsworth et al. (2016), organizational learning becomes collective learning, requiring social cohesion and activities [27]. In our study, clinical educators played a pivotal role in the successful implementation of SDL. Other studies, such as those of Rappolt et al. (2005), also emphasize the importance of dedicated staff and support for the successful implementation of SDL [28].

In our study, clinical educators supported and trained healthcare professionals through peer-to-peer learning in pairs. Practicing independently in pairs allowed for a relaxed exchange of reflection and feedback. Unlike regular training sessions where many colleagues observe, this approach enhanced psychological safety. Referring to Rudolph et al., our clinical educators, who act as instructors, view colleagues as individuals constructing meaning about their surroundings [29]. By engaging in dialogue and understanding their perspective, healthcare professionals demonstrated a heightened interest in learning.

Psychological safety means feeling secure to perform tasks without fear of missing out, sharing thoughts, and not facing punishment or rejection for speaking up or incomplete task performance [29]. Our study revealed that, following training care tasks with SDL, there was no difference in self-efficacy between those who scored high and those who scored low. This result was somewhat unexpected, and we believe it highlights a crucial point for managers: more experienced healthcare professionals don’t always perform better.

Interestingly, more healthcare professionals self-assess that they have the ability to lead patient care and involve the patients in care tasks, which we see as a prerequisite for taking responsibility for one’s own competence and ability to care for patients. In addition, the time for learning was adjusted so that it did not interfere with patient care. Thus, learning through SDL can contribute to maintaining the desired staffing level in the patient care while learning is ongoing, but it requires a culture of learning in the workplace. Aiken et al. (2002) provide evidence linking nursing staffing levels to the rescue of patients in life-threatening conditions, emphasizing nurses’ crucial role in early detection and timely interventions that save lives [30]. Future studies are needed to clarify SDL as a learning method for early detection of critically ill patients.

Post-training, a greater number of healthcare professionals reported an increased self-perceived ability to independently lead patient care. This enhanced sense of clinical autonomy was seen as contributing to more confident and safer interactions with patients in real-world care settings. Self-confident, healthcare professionals deliver safe care. It is the belief in one´s ability to get the job done, or self-efficacy, that must be fostered, even without a specific description of required nursing care [17, 31]. Embedding SDL as a part of CPD makes learning an integral part of the workplace culture with maintenance of motivation for learning among healthcare professionals [10].

Participants in our study viewed SDL as a means to attain a minimum common competence. Tønnessen et al. (2020) highlight considerations for developing a standard of safe, competent nursing care, emphasizing care provision based on patients’ needs and attention to fundamental needs to safeguard moral values [31]. We propose that SDL, when combined with other learning methods, contributes to achieving a minimum common competence. This supports healthcare professionals to adapt care to different settings and contexts, thereby meeting the requirements of reliable caregiving services. It is evident that more healthcare professionals perceive learning as an integral part of professional practice after training in care situations with SDL. While these findings provided valuable insights, they should be interpreted with caution until independently replicated in future research.

### Strengths and limitations

A strength of this study was the combination of methods that enabled us to cross-check if insights from interviews, also were reflected in the observations and survey results and vice versa. That also made us aware of aspects that would not have been captured using either observations or interviews only. Mixing methods also enabled us to look for relationships between quantitative measurements of abilities and qualitative expressions of experience, thus achieving integration of methods [32].A limitation was that the mixed-methods approach, combining interviews, observations, and surveys, was fully utilized only for the theme on the effects of continuing professional development. The other themes in the study relied solely on the interview data.

The researchers’ combined experience in qualitative and mixed methods research, along with author collaboration, influenced the study’s credibility. We recognized that our differing professional backgrounds—one as an intensive care nurse with a PhD and Associate Professor in caring science, and the other with a PhD in Technology and Social Change focused on healthcare innovation—could influence data collection and interpretation. To mitigate this, we employed ongoing reflexivity throughout the study, including continuous self-reflection, peer debriefing, and critical dialogue. This approach helped us remain aware of our positionality and potential biases, contributing to a transparent and trustworthy analytical process. Our interdisciplinary collaboration not only kept us focused on the research question and enabled us to approach the data from complementary perspectives, but also presented challenges in prioritizing insights, demanding continuous reflection to align with the study’s aim.

In the current study, one of the interviews was conducted with two managers present at the same interview. Our perception is that there are no limitations in the results because of this. Instead, it was a strength that the two managers could discuss their topics together.

A limitation of the study is the relatively small number of healthcare professionals who opted for repeated training and a study design that did not take a more longitudinal approach to examine the intervention’s potential long-term effects [33]. The challenge of recruiting healthcare professionals to embrace a new learning method, such as SDL, was evident. Given their vital role in providing comprehensive care to diverse patients, healthcare professionals must enhance their skills through evidence-based practice. A self-directed learning (SDL) approach to continuing professional development (CPD) can serve as a valuable complement other existing learning methods.

## Conclusions

In our study, all the interviewed groups (healthcare professionals, clinical educators and manager) emphasized the crucial role of clinical educators and educational coordinators in organizing and promoting learning within everyday healthcare settings, thereby enabling SDL. Healthcare professionals perceive SDL as a complementary learning method that enhances their self-efficacy.

Managers are responsible for prioritizing learning for their employees and for striving for a common minimum competence, ensuring that in healthcare professionals perform care tasks in accordance with evidence and guidelines. Establishing a culture of learning is a shared responsibility between managers and healthcare professionals.

Through self-directed learning (SDL), individuals gain important objective feedback from the mobile skills training system, that enhances their understanding of their own competence. For patients, the benefit lies in not being used as practice objects; instead, healthcare professionals first practice on mannequins. This dual benefit supports both individual development and workplace quality, as SDL conducted through peer-to-peer learning ensures accessible and continuous professional development for healthcare professionals. Moreover, peer-to-peer SDL encourages reflection on the learning process among healthcare professionals.

### Lessons for practice

* Clinical educators played a pivotal role in arranging SDL in a structured and inspiring way.* Clinical educators played a pivotal role in arranging SDL in a structured and inspiring way.

* Mobile skills training system in a clinical and peer-to-peer SDL setting supported CPD and reduced the perceived time healthcare professionals needed to be away from patient care.

* Clinical educators relied on crucial assistance from a clinical skills centre, which allowed them to plan, support, and follow-up on educational activities based on their workload and the learning needs of their colleagues.

* Managers and healthcare professionals, with sustained support from clinical skills centre educational coordinators, established a learning culture within the workplace. SDL is a complementary learning method that create a conducive and relaxed learning environment aligned with established evidence and guidelines across diverse care aspects.

## Supplementary Information

Supplementary Material 1.

Supplementary Material 2.

Supplementary Material 3.

Supplementary Material 4: Data structure and thematic map over experiences of what conditions and effects the use of a mobile educational system in a clinical and peer-to-peer SDL environment requires and provides. *SDL Self-Directed Learning. ** Researched using both qualitative (interview) and quantitative methods (survey and observation). *** CPD Continuing Professional Development. 

Supplementary Material 5: Perceived ability to engage the patient in the care task, before and after self-directed learning. 

## Data Availability

The data sets used and/or analysed during the current study will be available from the corresponding author in response to reasonable requests.

## Abbreviations

CPD: Continuing professional development
CPR: Cardiopulmonary resuscitation
SDL: Self-directed learning
