# Supplementary material for: A mixed methods study of self-directed learning in clinical practice using a mobile skills training system
Source: BMC Med Educ. 2025 Oct 29;25:1515. doi: 10.1186/s12909-025-08127-1 (PMC12570757; doi:10.1186/s12909-025-08127-1)
Supplement: Supplementary file 1 — Supplementary Material 1. [file 12909_2025_8127_MOESM1_ESM.docx]

**Supplemental material 1.**

**Checklist for Cardiopulmonary Resuscitation (CPR):**

*Answer alternatives for all questions:*

- Approved
- Partly performed
- Failed

1. The facilitator says loudly: "You have an apparently lifeless person in front of you, what do you do?"
   - Evaluate if the colleague: assesses signs of life, i.e., checks consciousness and breathing for a maximum of 10 seconds. In the assessment of breathing, a slight chin lift can be performed to try to create a clear airway.
2. The facilitator says: "During the assessment of signs of life, it becomes clear that the person is unconscious and has no breathing. What do you do?" Evaluate if the colleague: Alarms and starts CPR.
   - Information for the facilitator: if the participant does not start CPR on the mannequin but says that they alarm and start CPR, click on "partially performed" below.
3. Evaluate how the colleague asks for help, i.e., alarms.
   - Information for the facilitator: When the colleague says that the alarm is done, they have indirectly asked for help to retrieve a defibrillator and emergency equipment. The facilitator asks: "Do you know which telephone number to call depending on whether you are at work (internal alarm number 73111), in town, or at someone's home (112)?" It is the facilitator who ensures that defibrillator and emergency equipment are available for your practicing colleague.
4. Evaluate that the colleague starts CPR with 30 compressions in the center of the chest, 5-6 cm deep, at a rate of 100-120/min.
   - Information for the facilitator: you now let the colleague practice performing chest compressions and breaths for a while.
5. Evaluate that the colleague performs breaths after every 30th compression. Evaluate if the colleague performs the breath by blowing slowly until the chest rises, for about 1 second. That two breaths are performed in succession.
6. Evaluate that the colleague: Waits for the defibrillator's advice.
   - If advised to give a shock, the colleague should clearly warn so that no one touches the patient during analysis and when the shock is delivered.
7. The facilitator says: "A pharyngeal tube is available."
   - Evaluate that the colleague: measures the pharyngeal tube/nasal tube correctly and applies the tube correctly to secure a clear airway. Information for the facilitator, the size of the pharyngeal tube is measured by placing the tube at the center of the upper row of teeth to the jaw angle of the patient before it is placed in the patient's mouth.
8. Evaluate that the colleague: at some point says that oxygen at 10 liters/minute should be connected to a breathing mask or similar.

**Checklist for Urinary Catheterization:**
*Answer alternatives for all questions:*

- Approved
- Partly performed
- Failed

The practitioner shall now demonstrate how, with a structured approach, they perform catheterization of the bladder on a training model that represents the patient.

This shall be performed aseptically. (materials maintain their original degree of cleanliness)

1. Observe if your colleague gives information to the patient and checks ID.
2. Prepare and gather materials.
   - Observe if the colleague gathers:
     - Sterile material:
       - Sterile packaged catheter
       - Clean gloves
       - Hygiene underpad
       - Sterile catheterization set (forceps, sterile gloves, sterile compresses, kidney dish, sterile drapes, sterile swabs, small bowl)
     - Other material:
       - Soap and washcloths (Antiseptic agents should not be used)
       - Sodium chloride 9 mg/ml.
       - Anesthetic gel
       - 1, 10 mL syringe to fill the catheter balloon with sterile water, or glycerin mixture if a silicone catheter is used.
       - 10 mL sterile water for the catheter balloon - possibly glycerin mixture for silicone catheter.
       - Sodium chloride 9 mg/mL and irrigation syringe must be available.
       - Catheter valve and/or urine collection bag.
       - Fixation device.
3. Observe if the colleague disinfects the work surface.
4. Observe if the colleague performs thorough hand washing and disinfection of their hands.
5. Observe if the colleague puts on clean gloves and an apron.
6. Observe if the colleague works aseptically, i.e., maintains the sterile material sterile.
7. Observe if the colleague places a hygiene underpad under the patient.
8. Observe if the colleague performs thorough lower toilet with soap and water on the patient. (Washing correctly means washing from clean to unclean, keeping the clean clean)
9. Observe if the colleague removes dirty gloves and performs disinfection of their hands and puts on sterile gloves.
10. Observe if the colleague draws up the correct amount of anesthetic and anesthetizes correctly. (at least 10 ml for women and at least 20 ml for men) and waits for the effect for about 3-5 minutes.
11. Observe if the colleague inserts the urinary catheter correctly and considers asepsis/cleanliness.
12. Observe if the colleague checks for urine return in the catheter.
    - Information for the observer: The person inserting the urinary catheter must never inflate the catheter balloon if no urine return is obtained. The risk is then to inflate in the urethra.
13. Observe if the colleague inflates the catheter balloon with the correct amount and correct solution.
14. Observe if the colleague connects the correct type of urine collection bag in an aseptic manner.

**Checklist for Tracheostomy:***Answer alternatives for all questions:*

- Approved
- Partly performed
- Failed

1. The facilitator says: "There is an acute blockage in the patient's tracheostomy." Instruct the colleague to gather and describe what materials should be in the patient's immediate vicinity.
   - Evaluate if all materials are gathered: scissors, syringe (10 ml), syringe (2 ml), Sodium chloride 9 mg/ml, long nasal speculum (alternatively forceps or hooks), a spare cannula of the same type and size as the one the patient has and one in a smaller size, anesthetic ointment.
2. The facilitator says: "The materials are now gathered. You are now to continue performing the steps to remove the blockage in the patient's airway. How do you prepare yourself hygienically?"
   - Evaluate if the colleague: puts on a visor or protective glasses and a face mask, an apron, and gloves.
3. The facilitator says: "You have now gathered all the materials, what do you do now?"
   - The facilitator evaluates if the colleague alarms and acts quickly.
4. The facilitator says: "What do you do while waiting for help?"
   - Evaluate if the colleague removes the inner cannula and suctions away mucus.
5. The facilitator says: "The patient still has a blockage in the tracheostomy. How do you proceed?"
   - Evaluate if the colleague injects Sodium chloride 9 mg/mL, 2-3 mL, and suctions clean with a coarse suction catheter and repeats the procedure a few more times.
6. The facilitator says: "There is still no air passage, what do you do now?"
   - Evaluate if the colleague draws out all the air from the cuff with an empty syringe and cuts off the cannula band.
7. Evaluate if the colleague removes the tracheal cannula.
8. The facilitator says: "Now we have removed the tracheal cannula, how do you now maintain a clear airway?"
   - Evaluate if the colleague holds the tracheostomy open with a long nasal speculum and suctions clean in the trachea.
   - Evaluate if the colleague administers oxygen over the stoma.
