# Supplementary material for: A mixed methods study of self-directed learning in clinical practice using a mobile skills training system
Source: BMC Med Educ. 2025 Oct 29;25:1515. doi: 10.1186/s12909-025-08127-1 (PMC12570757; doi:10.1186/s12909-025-08127-1)
Supplement: Supplementary file 2 — Supplementary Material 2. [file 12909_2025_8127_MOESM2_ESM.docx]

**Supplemental material 2.**

**Survey Before Skills Training with Self-Directed Learning**

*Answer alternatives for all questions:*

- Strongly disagree
- Partially disagree
- Partially agree
- Strongly agree
- Don't know

1. In general, I feel confident in my professional skills regarding care procedures encountered in my daily work.
2. It is natural for healthcare professionals to occasionally make mistakes when performing care procedures.
3. I consider skills training to be an integral part of my professional practice.
4. I have reviewed the latest guidelines (e.g., Care Handbook) or performed a knowledge check before practicing skills.
5. I have the ability to involve the patient and adapt the care procedure to the patient's needs.
6. I can independently lead patient-centered care work.
7. What is your profession?
   - Registered Nurse (including specialist)
   - Assistant Nurse/Caregiver
   - Doctor
   - Physiotherapist
   - Other profession (please specify)
8. Have you previously received training in the specific skill area you are about to practice, apart from your basic professional education?
   - Yes
   - No

**Survey After Skills Training with Self-Directed Learning**

*Answer alternatives for all questions:*

- Strongly disagree
- Partially disagree
- Partially agree
- Strongly agree
- Don't know

1. After practicing, I feel more confident in my ability to perform care procedures encountered in my daily work (e.g., inserting a urinary catheter, taking venous samples, suctioning the airway via tracheostomy, maintaining and securing a clear airway, or performing chest compressions).
2. It is natural for healthcare professionals to occasionally make mistakes when performing care procedures.
3. I consider skills training to be an integral part of my professional practice.
4. Practicing skills with a colleague has influenced my confidence in performing patient care procedures.
5. I have the ability to involve the patient and adapt the care procedure to the patient’s needs.
6. I can independently lead patient-centered care work.
7. I have the skills to lead and advise a colleague in performing care procedures.
8. The training environment was appropriately designed for skills training.
9. The training material was well-suited for skills training.
10. The time allocated for training was appropriate for the content and learning objectives.
11. The information provided before the skills training was sufficient.
12. Following training, I feel capable of applying the skills I have learned in my professional practice.
13. Which clinic do you belong to?

- Surgical Clinic
- Orthopedic Clinic
- Medical Clinic
- Operating Clinic
- Another workplace (please specify)

1. How old are you?

- 19-30 years
- 31-40 years
- 41-50 years
- 51-60 years
- Over 60 years
- Prefer not to say

1. How long have you worked in the healthcare profession (regardless of employer and profession)?

- 1-5 years
- 6-10 years
- 11-15 years
- 16-20 years
- Over 20 years

1. What is your profession?

- Registered Nurse (including specialist nurse)
- Assistant Nurse/Caregiver/Child Caregiver
- Doctor
- Other profession (please specify)
