# Supplementary material for: A mixed methods study of self-directed learning in clinical practice using a mobile skills training system
Source: BMC Med Educ. 2025 Oct 29;25:1515. doi: 10.1186/s12909-025-08127-1 (PMC12570757; doi:10.1186/s12909-025-08127-1)
Supplement: Supplementary file 3 — Supplementary Material 3. [file 12909_2025_8127_MOESM3_ESM.docx]

**Supplemental material 3. Sub them, codes and example of quotations from the interviews.**

|  | |  |
| --- | --- | --- |
| **Sub theme** | **Example of codes** | **Example of quotations** (translated from Swedish) |
| **Leadership required for learning** | Responsibility for creating a  learning culture Integrating learning into clinical practice | - If I put it (learning) in focus and say, 'This is something we must do'... It becomes such a culture in a workplace if I also prioritize it (learning). - I think a lot also lies in personal responsibility. Everyone has a personal responsibility to improve and keep up with developments in care practice, and then, of course, the Region also provides valuable support. One needs to develop one's competence, learn more and be open to it. - Managing all training alone becomes too challenging for a manager. It is therefore crucial to have healthcare professionals who take initiatives and genuinely believe in the value of this endeavour. This ensures that it doesn't become overwhelming for everyone, including ourselves, and that we don't lose momentum. - My experience with competency cards (online learning) is that they are very good. But it's also up to oneself because it's possible to cheat through them, unfortunately. - It really requires someone who is like, 'now let's take hold of it and do it. |
| **Conditions for learning** | Prioritize learning/training  Organize training | - There's hardly ever enough time for everything, but it's essential to make time. Given my prioritization of education and developmental matters, it's evident that education must take precedence. It not only nourishes us but also fosters increased motivation, attraction, curiosity, joy, and pride. - Yes, but everyone is unique, some may need to practice ten times, while others may only need to do it twice. It's crucial to identify the factors hindering learning. - I think the purpose of self-directed learning from the clinical management has been to reduce the time that we instructors spend on our training. So, if some group of colleagues would need an instructor, it's not certain that we're allowed to leave (to organize for them). - That said, simplicity is everything and I´m glad we´re at it. We had an ambition, we came to you (Clinical Skills centre), we looked, we had a plan, and then suddenly, here we are |
| **Effects of CPD*** | Relevance for the individual Relevancefor the patient and  the workplace | - So, it becomes a very good learning experience, that you can do it several times until you get a percentage that feels good. - I think it feels very appropriate to be in pairs. You can get good help from each other. It leads to good discussions... or well, reflections and feedback. Now you have something to lean on a bit more, so concretely. - People feel more secure practicing on a mannequin first, completing all the steps before transitioning to human patients. - I would say that it has to do with patient safety. So, this (self-directed learning) makes us safer and more secure. Yes, and what does a patient need, a sick person needs… nothing but a safe and warm and knowledgeable staff around him. And I think these simplistic learning methods are helping us, so I´m really happy. - So, I think that the biggest benefit for the patient is that you are confident and secure. And you know that if you get a question... You should be able to answer questions if the patient asks you, 'why are you doing it this way?' It has to be professional; you know. So, there are only advantages to it (self-directed learning). - It contributes to ensuring that everyone performs a task in the same way, that it is evidence-based, and that it is based on a platform where the information is derived from evidence-based knowledge. |
| Suggestions for improving training methods | Technical and communication  development  Empowering clinical educators to enhance learning | - I learn better, for example, if... when I'm on the internet reading, I highlight everything, and then I have it read aloud. I think, since there are many with dyslexia. Am I going to read all of this? |
|  |  |  |

*CPD; Continuing Professional Development.
