# Supplementary figures and images for: A mixed methods study of self-directed learning in clinical practice using a mobile skills training system
Source: BMC Med Educ. 2025 Oct 29;25:1515. doi: 10.1186/s12909-025-08127-1 (PMC12570757; doi:10.1186/s12909-025-08127-1)

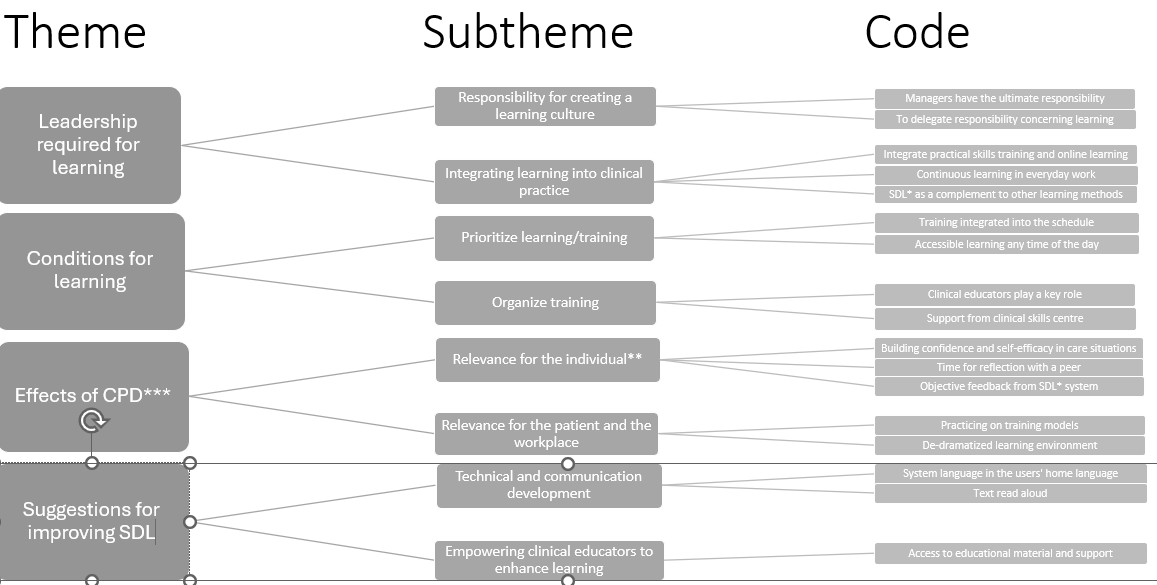

Supplement: Supplementary file 4 — Supplementary Material 4: Data structure and thematic map over experiences of what conditions and effects the use of a mobile educational system in a clinical and peer-to-peer SDL environment requires and provides. *SDL Self-Directed Learning. ** Researched using both qualitative (interview) and quantitative methods (survey and observation). *** CPD Continuing Professional Development. [file 12909_2025_8127_MOESM4_ESM.png]
