# Supplementary material for: A mixed methods study of self-directed learning in clinical practice using a mobile skills training system
Source: BMC Med Educ. 2025 Oct 29;25:1515. doi: 10.1186/s12909-025-08127-1 (PMC12570757; doi:10.1186/s12909-025-08127-1)
Supplement: Supplementary file 5 — Supplementary Material 5: Perceived ability to engage the patient in the care task, before and after self-directed learning. [file 12909_2025_8127_MOESM5_ESM.docx]

Additional file 1. Perceived ability to engage the patient in the care task, before and after self-directed learning.

|  |  | **^Before^** | **^After^** |
| --- | --- | --- | --- |
|  | **^Do not know^** | ^1 (1.3)^ | ^0 (0)^ |
|  | **^Not true^** | ^2 (2.6)^ | ^3 (3.8)^ |
|  | **^Partly true^** | ^20 (25.6)^ | ^17 (21.8)^ |
|  | **^True^** | ^55 (70.5)^ | ^58 (74.4)^ |
|  | **^Total N^** | ^78 (100)^ | ^78 (100)^ |

Cross tables of self-assessments displaying changes before and after self-directed learning. Changes are read row-wise left to right, e.g. participant reporting “not true” before practicing and how they report after practice
